# One-dimensional differential Hardy inequality

**DOI:** 10.1186/s13660-017-1293-3

**Published:** 2017-01-17

**Authors:** Aigerim Kalybay

**Affiliations:** 1 KIMEP University, Abai Ave. 4, Almaty, 050010 Kazakhstan; 2 L.N. Gumilyov Eurasian National University, Munaytpasov St. 5, Astana, 010008 Kazakhstan

**Keywords:** 26D10, 47B38, inequality, Hardy-type inequality, weight, differential operator

## Abstract

We establish necessary and sufficient conditions for the one-dimensional differential Hardy inequality to hold, including the overdetermined case. The solution is given in terms different from those of the known results. Moreover, the least constant for this inequality is estimated.

## Introduction

Let $I=(a,b)$, $-\infty\leq a< b\leq\infty$, $1\leq p, q\leq\infty$, $\frac{1}{p}+\frac{1}{p'}=1$, and $\frac{1}{q}+\frac{1}{q'}=1$. Let $u\geq0$ and $\rho>0$ be weight functions such that $u\in L_{q}^{\mathrm {loc}}$ and $\rho^{-1}\equiv\frac{1}{\rho}\in L_{p'}^{\mathrm {loc}}$, where $L_{p}\equiv L_{p}(I)$ stands for the space of measurable functions *f* on *I* with finite norm

$$\Vert f\Vert _{p}=\left \{ \textstyle\begin{array}{l@{\quad}l} (\int_{a}^{b} \vert f(t)\vert ^{p}\,dt )^{\frac{1}{p}}, & 1\leq p < \infty, \\ \operatorname {ess\,sup}_{t\in I}\vert f(t)\vert , & p=\infty. \end{array}\displaystyle \right . $$

Let $\operatorname {AC}(I)$ be the set of all functions locally absolutely continuous on *I*. Let $\overset{\circ }{\mathrm{AC}}(I)$ be the set of functions from $\operatorname {AC}(I)$ with compact supports on *I*.

We consider the following Hardy inequality in the differential form

1 $${∥uf∥}_{q}\leq C{∥\rho f^{\prime }∥}_{p},\;f\in \overset{\circ }{\mathrm{AC}}(I).$$

In [1] it is shown that if

2 $$ \int_{a}^{c} \rho^{-p'}(s)\,ds=\infty\quad \mbox{and}\quad \int_{c}^{b} \rho^{-p'}(s)\, ds=\infty $$

for some $c\in I$, then inequality (1) does not hold. In the case

3 $$ \int_{a}^{c} \rho^{-p'}(s)\,ds< \infty\quad \mbox{and}\quad \int_{c}^{b} \rho^{-p'}(s)\, ds=\infty $$

or

4 $$ \int_{a}^{c} \rho^{-p'}(s)\,ds=\infty\quad \mbox{and}\quad \int_{c}^{b} \rho^{-p'}(s)\, ds< \infty, $$

then inequality (1) is satisfied for all functions $f\in \operatorname {AC}(I)$ such that $f(a)=0$ or $f(b)=0$, respectively. For example, in case (3), it is equivalent to the weighted integral Hardy inequality (see [1])

5 $$ \biggl( \int_{a}^{b}\biggl\vert u(x) \int_{a}^{x}f(s)\,ds\biggr\vert ^{q}\, dx \biggr)^{\frac{1}{q}}\leq C \biggl( \int_{a}^{b}\bigl\vert v(t)f(t)\bigr\vert ^{p}\,dt \biggr)^{\frac{1}{p'}}, $$

which has been studied for all values of the parameters $0< p,q\leq \infty$ (see [2, 3], and [4]).

In [1] it is also shown that in the last case

6 $$ \bigl\Vert \rho^{-1}\bigr\Vert _{p'}< \infty, $$

inequality (1) is satisfied for all functions $f\in \operatorname {AC}(I)$ such that $f(a)=0$ and $f(b)=0$, that is, it is an overdetermined case. This case is studied in [1, 2], and [4].

In the present work, for $1\leq p\leq q\leq\infty$, we establish a criterion for the validity of inequality (1) with an estimate of the type

7 $$ B(u,\rho)\leq C\leq C_{1} B(u,\rho) $$

for the least constant *C* in (1), where $B(u,\rho)$ is some functional depending on *u* and *ρ*. Moreover, we present a calculation formula for the least value of $C_{1}$ and its two-sided estimate.

We suppose that only condition (2) does not hold. In case (3) or (4), our criterion coincides with the well-known Muckenhoupt result. However, our upper estimate in (7) is worse than the known one (see [2–4], and Remark 3.2 further). In case (6), our criterion is given in terms different from those in [1, 2], and [4]. The terms in [4] are close to ours, but the comparison analysis shows that our results and an estimate of type (7) are better than in [4] (see Remark 3.3).

At the end of the paper, we find a criterion for the compactness of the set $M=\{uf:f\in \overset{\circ }{\mathrm{AC}}(I),{∥\rho f^{\prime }∥}_{p}\leq 1\}$ in $L_{q}(I)$.

## Auxiliary statements

### Lemma 2.1

*Let* $1< q<\infty$ *and* $\varphi(\lambda)=\frac{\lambda^{q}}{\lambda ^{q}-1}-\frac{1}{\lambda-1}$, $\lambda>1$. *There exists a point* $\lambda _{1}\equiv\lambda_{1}(q)$ *such that*

8 $$ \lambda_{1}(2)=\frac{1+\sqrt{5}}{2},\quad\quad \frac {2q}{q+1}< \lambda_{1}(q)< \min\{q,2\} \quad \textit{for } q\neq2, $$

*and*

9 $$ \varphi(\lambda_{1})=\frac{\lambda_{1}^{q}}{\lambda _{1}^{q}-1}- \frac{1}{\lambda_{1}-1}=0. $$

*In addition*, $\frac{\lambda^{q}}{\lambda^{q}-1}<\frac{1}{\lambda-1}$ *for* $1<\lambda<\lambda_{1}$ *and* $\frac{\lambda^{q}}{\lambda^{q}-1}>\frac{1}{\lambda-1}$ *for* $\lambda>\lambda_{1}$.

### Proof

Since

$$\varphi(\lambda)=\frac{1}{(\lambda^{q}-1)(\lambda-1)}\bigl(\lambda ^{q+1}-2 \lambda^{q}+1\bigr), $$

the sign of the function *φ* is defined by the value of the function $d(\lambda)=\lambda^{q+1}-2\lambda^{q}+1$. Moreover, $\varphi (\lambda)=0$, $\lambda>1$, if and only if $d(\lambda)=0$.

For $q=2$, we have $d(\lambda)=\lambda^{3}-2\lambda^{2}+1=(\lambda -1)(\lambda^{2}-\lambda-1)$. This means that $d(\lambda)=0$ for $\lambda =\frac{1+\sqrt{5}}{2}$.

Let $q\neq2$. Let $\lambda=1+\varepsilon$, $\varepsilon>0$. Using the Lagrange finite-increment formula, we have

$$\varphi(\lambda)=\frac{(1+\varepsilon)^{q}}{(1+\varepsilon)^{q}-1}-\frac {1}{\varepsilon}\geq \frac{(1+\varepsilon)^{q}}{q\varepsilon(1+\varepsilon)^{q-1}}- \frac {1}{\varepsilon}=\frac{1}{q\varepsilon}\bigl(\varepsilon-(q-1)\bigr). $$

This gives that $\varphi(\lambda)=\varphi(1+\varepsilon)\geq0$ for $\varepsilon\geq q-1$, that is, $\varphi(\lambda)>0$ for $\lambda>q$.

Let us find an extremum of the function *d* for $\lambda>1$. We have that $d'(\lambda)=(q+1)\lambda^{q}-2q\lambda^{q-1}=\lambda ^{q-1}((q+1)\lambda-2q)$. This gives that $d'(\lambda)=0$ for $\lambda =\frac{2q}{q+1}$, $d'(\lambda)>0$ for $\lambda>\frac{2q}{q+1}$, and $d'(\lambda)<0$ for $1<\lambda<\frac{2q}{q+1}$. Therefore, the function $d(\lambda)$ decreases for $1<\lambda\leq\frac{2q}{q+1}$ and increases for $\lambda>\frac{2q}{q+1}$. Moreover, it has a minimum at $\frac {2q}{q+1}$. Since $d(2)=1>0$, $d(\lambda)>0$ for $\lambda\geq2$. Hence, $\varphi(\lambda)>0$ for $\lambda>2$.

Thus,

10 $$ \varphi(\lambda)>0 \quad \mbox{for } \lambda>\min\{ q,2\}. $$

Since $d(1)=0$ and *d* has a minimum at $\frac{2q}{q+1}$, it follows that $d (\frac{2q}{q+1} )<0$ for $\lambda>1$ and $d(\lambda )<0$ for $1<\lambda\leq\frac{2q}{q+1}$. Therefore, $\varphi (\frac {2q}{q+1} )<0$, and

11 $$ \varphi(\lambda)< 0 \quad \mbox{for } 1< \lambda\leq \frac{2q}{q+1}. $$

In view of the continuity of *φ* for $\lambda>1$, from (10) and (11) there follows the existence of a point that satisfies (8) and (9).

The last statement of Lemma 2.1 follows from the intersection of graphs of two decreasing and concave upward functions $\frac{\lambda ^{q}}{\lambda^{q}-1}$ and $\frac{1}{\lambda-1}$ at the point $\lambda =\lambda_{1}$. The proof of Lemma 2.1 is complete. □

### Lemma 2.2

*Let* $1< q<\infty$ *and* $f(\lambda)=\frac{\lambda(\lambda^{q}-1)^{\frac {1}{q}}}{\lambda-1}$, $\lambda>1$. *Then*

12 $$ f\bigl(\lambda_{1}(2)\bigr)= \biggl(\frac{3\sqrt{5}+7}{\sqrt{5}-1} \biggr)^{\frac{1}{2}}, \quad\quad \inf_{\lambda>1}f(\lambda)=f( \lambda_{1})= \frac{\lambda_{1}^{2}}{(\lambda_{1}-1)^{\frac{1}{q'}}}, $$

*and*, *for* $q\neq2$, *we have the estimate*

13 $$ \gamma_{0}< f(\lambda_{1})< \min\{ \gamma_{1},\gamma_{2},4\}, $$

*where* $\gamma_{0}=\frac{2q}{q+1} (\frac{2q}{q+1} )^{\frac {1}{q}} (\frac{2q}{q-1} )^{\frac{1}{q'}}$, $\gamma_{1}=\frac {2q}{q+1}q^{\frac{1}{q}} (\frac{2q}{q-1} )^{\frac{1}{q'}}$, *and* $\gamma_{2}=qq^{\frac{1}{q}} (q' )^{\frac{1}{q'}}$.

### Proof

By Lemma 2.1 for $q=2$ we have $\lambda_{1}(2)=\frac{1+\sqrt {5}}{2}$, so that $f(\lambda_{1}(2))= (\frac{3\sqrt{5}+7}{\sqrt {5}-1} )^{\frac{1}{2}}$.

Let $q\neq2$. The function *f* is continuous when $\lambda>1$, and $\lim_{\lambda\rightarrow1+}f(\lambda)=\infty$ and $\lim_{\lambda \rightarrow\infty}f(\lambda)=\infty$. Therefore, it has a minimum. Since $f'(\lambda)=\frac{(\lambda^{q}-1)^{\frac{1}{q}}}{\lambda-1} (\frac {\lambda^{q}}{\lambda^{q}-1}-\frac{1}{\lambda-1} )$, by Lemma 2.1 we have that $f'(\lambda_{1})=0$, $f'(\lambda)<0$ for $1<\lambda<\lambda _{1}$, and $f'(\lambda)>0$ for $\lambda>\lambda_{1}$, that is, the function *f* decreases for $1<\lambda<\lambda_{1}$, increases for $\lambda>\lambda _{1}$, and has a minimum at $\lambda=\lambda_{1}$. Thus, $\inf_{\lambda >1}f(\lambda)=f(\lambda_{1})$. Again by Lemma 2.1 we have that $\frac {\lambda_{1}^{q}}{\lambda_{1}^{q}-1}=\frac{1}{\lambda_{1}-1}$. Substituting this equality into the expression of $f(\lambda_{1})$, we get (12).

The function $g(x)=\frac{\lambda^{2}}{(\lambda-1)^{\frac{1}{q'}}}$ has a minimum at the point $\lambda=\frac{2q}{q+1}$. Therefore,

14 $$ f(\lambda_{1})=g(\lambda_{1})>g \biggl( \frac {2q}{q+1} \biggr)= \frac{2q}{q+1} \biggl(\frac{2q}{q+1} \biggr)^{\frac{1}{q}} \biggl(\frac {2q}{q-1} \biggr)^{\frac{1}{q'}}. $$

By Lemma 2.1 we have that $\frac{2q}{q+1}<\lambda_{1}<\min\{q,2\}$. Hence,

15 $$ f(\lambda_{1})< \min \biggl\{ f \biggl(\frac {2q}{q+1} \biggr), f(q),f(2) \biggr\} . $$

It is easy to see that $f(2)<4$. Since $\lambda_{1}< q$, we have $\frac {q^{q}}{q^{q}-1}>\frac{1}{q-1}$ or $q(q-1)^{\frac{1}{q}}>(q^{q}-1)^{\frac {1}{q}}$. Therefore,

16 $$ f(q)=\frac{q(q^{q}-1)^{\frac{1}{q}}}{q-1}< \frac {q^{2}}{(q-1)^{\frac{1}{q'}}}=qq^{\frac{1}{q}} \bigl(q'\bigr)^{\frac{1}{q'}}. $$

Let us estimate $f (\frac{2q}{q+1} )$:

17 $$ \begin{aligned}[b] f \biggl(\frac{2q}{q+1} \biggr)&=\frac{2q}{q-1} \biggl[ \biggl(1+ \frac {q-1}{q+1} \biggr)^{q}-1 \biggr]^{\frac{1}{q}}\leq \frac{2q}{q-1} \biggl[q\frac{q-1}{q+1} \biggl(\frac{2q}{q+1} \biggr)^{q-1} \biggr]^{\frac{1}{q}} \\ &= \frac{2q}{q-1}q^{\frac{1}{q}} \biggl(\frac{q-1}{q+1} \biggr)^{\frac{1}{q}} \biggl(\frac{2q}{q+1} \biggr)^{\frac{1}{q'}}= \frac{2q}{q+1}q^{\frac{1}{q}} \biggl(\frac{2q}{q-1} \biggr)^{\frac{1}{q'}}. \end{aligned} $$

From (14), (15), (16), and (17), taking into account that $f(2)<4$, we have (13). The proof of Lemma 2.2 is complete. □

## Main results

Let $a< c< b$, $d=d(a,c,b)=\min\{c-a, b-c\}$. Assume that

$$\begin{aligned}& A_{p,q}(c)=\sup_{0< h< d}\frac{\Vert u\Vert _{q,(c-h,c+h)}}{ (\Vert \rho^{-1}\Vert ^{-p}_{p',(a,c-h)} +\Vert \rho^{-1}\Vert ^{-p}_{p',(c+h,b)} )^{\frac {1}{p}}}, \quad 1\leq p< \infty, \\& A_{p,q}(c)=\sup_{0< h< d}\frac{\Vert u\Vert _{q,(c-h,c+h)}}{\Vert \rho^{-1}\Vert ^{-1}_{1,(a,c-h)} +\Vert \rho^{-1}\Vert ^{-1}_{1,(c+h,b)}}, \quad p=\infty, \end{aligned}$$

and

$$A_{p,q}=\sup_{c\in I}A_{p,q}(c). $$

### Theorem 3.1

*Let* $1\leq p\leq q<\infty$. *Inequality* (1) *holds if and only if* $A_{p,q}<\infty$. *Moreover*, *for the least constant* *C* *in* (1), *we have the estimate*

18 $$ A_{p,q}\leq C\leq f(\lambda_{1})A_{p,q}. $$

*In turn*, *for* $f(\lambda_{1})$, *by Lemma* 2.2 *we have* $f(\lambda _{1}(2))= (\frac{3\sqrt{5}+7}{\sqrt{5}-1} )^{\frac{1}{2}}$ *and the estimate* $\gamma_{0}< f(\lambda_{1})<\min\{\gamma_{1},\gamma_{2},4\}$ *for* $q\neq2$.

### Proof

Necessity. Let inequality (1) hold with the least constant $C>0$.

Suppose that $1< p<\infty$. Let $c\in I$, $0< h< d$, and $a<\alpha <c-h<c+h<\beta<b$. We introduce the following function:

$$f_{c,h}(t)=\left \{ \textstyle\begin{array}{l@{\quad}l} \int_{\alpha}^{t}\rho^{-p'}(s)\,ds (\int_{\alpha}^{c-h} \rho ^{-p'}(s)\,ds )^{-1}, & \alpha\leq t\leq c-h, \\ 1, & c-h\leq t\leq c+h,\\ \int_{t}^{\beta}\rho^{-p'}(s)\,ds (\int_{c+h}^{\beta} \rho ^{-p'}(s)\,ds )^{-1} , & c+h\leq t\leq\beta, \\ 0, & t\in I\setminus(\alpha,\beta). \end{array}\displaystyle \right . $$

It is obvious that $f_{c,h}\in \overset{\circ }{\mathrm{AC}}(I)$. Substituting $f_{c,h}$ into (1), we get

$$\Vert u\Vert _{q,(c-h,c+h)}\leq C \bigl(\bigl\Vert \rho ^{-1} \bigr\Vert ^{-p}_{p',(\alpha,c-h)} +\bigl\Vert \rho^{-1}\bigr\Vert ^{-p}_{p',(c+h,\beta)} \bigr)^{\frac{1}{p}}. $$

From the last inequality, taking into account that its left-hand side does not depend on *α*, *β* such that $a<\alpha<\beta<b$, we have

19 $$ \Vert u\Vert _{q,(c-h,c+h)}\leq C \bigl(\bigl\Vert \rho^{-1}\bigr\Vert ^{-p}_{p',(a,c-h)} +\bigl\Vert \rho^{-1}\bigr\Vert ^{-p}_{p',(c+h,b)} \bigr)^{\frac{1}{p}} $$

for all $c\in I$ and $0< h< d$.

In the case $p=1$, we construct *f* in the following way. Let numbers *c* and *h* be defined as before, $\delta>0$, and $a< x-\delta< x+\delta \leq c-h< c+h\leq y-\delta< y+\delta< b$. Assume that

$$f_{c,h}(t)=\left \{ \textstyle\begin{array}{l@{\quad}l} \int_{x-\delta}^{t}\rho^{-1}(s)\,ds (\int_{x-\delta}^{x+\delta} \rho ^{-1}(s)\,ds )^{-1}, & x-\delta\leq t\leq x+\delta, \\ 1, & x+\delta\leq t\leq y-\delta,\\ \int_{t}^{y+\delta}\rho^{-1}(s)\,ds (\int_{y-\delta}^{y+\delta} \rho^{-1}(s)\,ds )^{-1} , & y-\delta\leq t\leq y+\delta, \\ 0, & t\in I\setminus(x-\delta,y+\delta). \end{array}\displaystyle \right . $$

Substituting $f_{c,h}$ into (1), we get

$$\Vert u\Vert _{q,(x+\delta,y-\delta)}\leq C \biggl[ \biggl(\frac {1}{2\delta} \int_{x-\delta}^{x+\delta} \rho^{-1}(s)\,ds \biggr)^{-1}+ \biggl(\frac{1}{2\delta} \int_{y-\delta}^{y+\delta} \rho^{-1}(s)\, ds \biggr)^{-1} \biggr]. $$

Taking the limit in this inequality as $\delta\rightarrow0$, we get

$$\Vert u\Vert _{q,(x,y)}\leq C \bigl(\rho(x)+\rho(y) \bigr) $$

for almost all $x:(a< x\leq c-h)$ and almost all $y:(c+h\leq y< b)$.

For $\alpha>1$, there exist points $x:(a< x\leq c-h)$ and $y:(c+h\leq y< b)$ such that

$$\frac{\Vert \rho^{-1}\Vert _{\infty,(a,c-h)}}{\alpha}\leq \rho^{-1}(x) \quad \mbox{and} \quad \frac{\Vert \rho^{-1}\Vert _{\infty,(c+h,b)}}{\alpha}\leq \rho^{-1}(y). $$

Then

$$\Vert u\Vert _{q,(x,y)}\leq\alpha C \bigl[\bigl(\bigl\Vert \rho ^{-1}\bigr\Vert _{\infty,(a,c-h)}\bigr)^{-1}+ \bigl(\bigl\Vert \rho^{-1}\bigr\Vert _{\infty,(c+h,b)}\bigr)^{-1} \bigr]. $$

This, together with $\Vert u\Vert _{q,(x,y)}\geq \Vert u\Vert _{q,(c-h,c+h)}$, yields that

$$\Vert u\Vert _{q,(c-h,c+h)}\leq\alpha C \bigl[\bigl\Vert \rho ^{-1}\bigr\Vert _{\infty,(a,c-h)}^{-1}+ \bigl\Vert \rho^{-1}\bigr\Vert _{\infty,(c+h,b)}^{-1} \bigr]. $$

Since the left-hand side of this inequality does not depend on $\alpha >1$, letting $\alpha\rightarrow1$, we get (19) for $p=1$. Thus, for all $1\leq p\leq q<\infty$, we have that

20 $$ A_{p,q}\leq C. $$

Sufficiency. Let $A_{p,q}<\infty$ be correct. Let *f* be a nontrivial function from $\overset{\circ }{\mathrm{AC}}(I)$. Without loss of generality, we assume that $f\geq0$. Let $\lambda>1$. For any integer *k*, we assume that $T_{k}=\{t\in I:f(t)>\lambda^{k}\}$, $\Delta T_{k}=T_{k}\setminus T_{k+1}$. Due to the boundedness of the function *f*, there exists an integer $n=n(f)$ such that $T_{n}\neq\emptyset$ and $T_{n+1}=\emptyset$. It is obvious that $I=\bigcup_{k\leq n}T_{k}=\bigcup_{k\leq n}\Delta T_{k}$. The set $T_{k}$ is open. Therefore, there exists a family of mutually disjoint intervals $\{J_{j}^{k}\}$, $J_{j}^{k}=(c_{j}^{k},d_{j}^{k})$, such that $T_{k}=\bigcup_{j}J_{j}^{k}$. For $n-1\geq k>-\infty$, we assume that $M_{j}^{k}=T_{k+1}\cap J_{j}^{k}$. For $M_{j}^{k}\neq\emptyset$, we define $\alpha _{j}^{k}=\inf M_{j}^{k}$ and $\beta_{j}^{k}=\sup M_{j}^{k}$. Then

21 $$ T_{k+1}\subset\bigcup_{j} \bigl(\alpha_{j}^{k},\beta _{j}^{k} \bigr) \quad \mbox{and} \quad \Delta T_{k}\supset\bigcup _{j}\bigl[\bigl(c_{j}^{k}, \alpha_{j}^{k}\bigr)\cup \bigl(\beta_{j}^{k}, d_{j}^{k}\bigr)\bigr]. $$

In view of the continuity of the function *f*, we get that $f(\alpha _{j}^{k})=f(\beta_{j}^{k})=\lambda^{k+1}$ and $f(c_{j}^{k})=f(d_{j}^{k})=\lambda^{k}$. Hence,

22 $$ \lambda^{k}(\lambda-1)=\lambda^{k+1}-\lambda ^{k}= \int_{c_{j}^{k}}^{\alpha_{j}^{k}}f'(s)\,ds=- \int_{\beta_{j}^{k}}^{d_{j}^{k}}f'(s)\,ds. $$

From (22) by Hölder’s inequality we have that

23 $$\begin{aligned} &\lambda^{kp}\bigl\Vert \rho^{-1}\bigr\Vert _{p',(c_{j}^{k},\alpha_{j}^{k})}^{-p}\leq\frac{\Vert \rho f'\Vert _{p,(c_{j}^{k},\alpha_{j}^{k})}^{p}}{(\lambda-1)^{p}}, \end{aligned}$$

24 $$\begin{aligned} &\lambda^{kp}\bigl\Vert \rho^{-1}\bigr\Vert _{p',(\beta_{j}^{k},d_{j}^{k})}^{-p}\leq\frac{\Vert \rho f'\Vert _{p,(\beta_{j}^{k},d_{j}^{k})}^{p}}{(\lambda-1)^{p}}. \end{aligned}$$

In view of $f(t)<\lambda^{k+1}$ for $t\in\Delta T_{k}$ and $\lambda ^{qk}=(1-\lambda^{-q})\sum_{i\leq k}\lambda^{qi}$, we have that

$$\begin{aligned} \Vert uf\Vert _{q}^{q}&=\sum _{k\leq n-1}\Vert uf\Vert _{q,\Delta T_{k+1}}^{q}\leq \sum _{k}\lambda^{q(k+2)}\Vert u\Vert _{q,\Delta T_{k+1}}^{q} \\ &\leq\lambda^{q}\bigl(\lambda^{q}-1\bigr) \sum _{k}\Vert u\Vert _{q,\Delta T_{k+1}}^{q}\sum _{i\leq k}\lambda^{qi}= \lambda^{q} \bigl(\lambda^{q}-1\bigr)\sum_{i} \lambda^{qi} \sum_{k\geq i}\Vert u\Vert _{q,\Delta T_{k+1}}^{q} \\ &= \lambda^{q}\bigl(\lambda^{q}-1\bigr)\sum _{i}\lambda^{qi} \Vert u\Vert _{q,T_{i+1}}^{q} \end{aligned}$$

(by (21))

$$\leq \lambda^{q}\bigl(\lambda^{q}-1\bigr)\sum _{i}\lambda^{qi} \sum_{j} \int_{\alpha_{j}^{i}}^{\beta_{j}^{i}}u^{q}(s)\,ds= \lambda^{q}\bigl(\lambda^{q}-1\bigr)\sum _{i}\lambda^{qi} \sum_{j} \Vert u\Vert _{q,(\alpha_{j}^{i},\beta_{j}^{i})}^{q}. $$

Since $A_{p,q}<\infty$, from (23) and (24), taking into account that $\frac{q}{p}\geq1$, this gives

$$\begin{aligned} \Vert uf\Vert _{q}^{q}&\leq\lambda^{q}\bigl( \lambda^{q}-1\bigr) A_{p,q}^{q}\sum _{i}\sum_{j} \bigl( \lambda^{pi}\bigl\Vert \rho^{-1}\bigr\Vert _{p',(c_{j}^{i},\alpha _{j}^{i})}^{-p}+\lambda^{pi}\bigl\Vert \rho^{-1}\bigr\Vert _{p',(\beta _{j}^{i},d_{j}^{i})}^{-p} \bigr)^{\frac{q}{p}} \\ &\leq\frac{\lambda^{q}(\lambda^{q}-1)}{(\lambda-1)^{q}} A_{p,q}^{q} \biggl(\sum _{i}\sum_{j} \bigl(\bigl\Vert \rho f'\bigr\Vert _{p,(c_{j}^{i},\alpha_{j}^{i})}^{p}+\bigl\Vert \rho f'\bigr\Vert _{p,(\beta_{j}^{i},d_{j}^{i})}^{p} \bigr) \biggr)^{\frac{q}{p}} \end{aligned}$$

(by the second relation from (21))

$$\leq\frac{\lambda^{q}(\lambda^{q}-1)}{(\lambda-1)^{q}} A_{p,q}^{q} \biggl(\sum _{i} \bigl\Vert \rho f'\bigr\Vert _{p,\Delta T_{i}}^{p} \biggr)^{\frac {q}{p}}\leq\frac{\lambda^{q}(\lambda^{q}-1)}{(\lambda-1)^{q}} A_{p,q}^{q} \bigl\Vert \rho f'\bigr\Vert _{p}^{q}. $$

Therefore,

25 $$ \Vert uf\Vert _{q}\leq\frac{\lambda (\lambda^{q}-1)^{\frac{1}{q}}}{(\lambda-1)} A_{p,q} \bigl\Vert \rho f'\bigr\Vert _{p}. $$

Since the left-hand side of this inequality does not depend on $\lambda >1$, by Lemma 2.2 we have

$$\Vert uf\Vert _{q}\leq f(\lambda_{1}) A_{p,q} \bigl\Vert \rho f'\bigr\Vert _{p}, $$

that is, inequality (1) holds with the estimate $C\leq f(\lambda _{1}) A_{p,q}$ for the least constant *C* in (1). This fact, together with (20), gives (18). The proof of Theorem 3.1 is complete. □

### Remark 3.1

Let us notice that in [5], for inequality (1), an estimate of the type (18) has been obtained in the case $1< q=p<\infty$.

### Remark 3.2

If (3) or (4) is correct, then by Theorem 3.1 there follows the correctness of the corresponding integral Hardy inequality (see [1]). For example, if (3) holds, then

$$A_{p,q}=\sup_{z\in I} \biggl( \int_{z}^{b}u^{q}(x)\,dx \biggr)^{\frac {1}{q}} \biggl( \int_{a}^{z}v^{-p'}(s)\,ds \biggr)^{\frac{1}{p'}}, $$

where the condition $A_{p,q}<\infty$ coincides with the Muckenhoupt condition (see [3]), and inequality (1) is equivalent to the integral Hardy inequality (5). However, our upper estimate in (7) is worse than that in the known result (see e.g. [3], Thm. 5). For example, in case (3) with $p=q=2$, from (12) we have $A_{p,q}\leq C\leq (\frac{3\sqrt{5}+7}{\sqrt {5}-1} )^{\frac{1}{2}}A_{p,q}\approx3.33 A_{p,q}$, but from Theorem 5 of [3] it follows that $A_{p,q}\leq C\leq2A_{p,q}$.

### Theorem 3.2

*Let* $1=p=q$. *Inequality* (1) *holds if and only if* $A_{p,q}<\infty$. *Moreover*, $A_{p,q}=C$, *where* *C* *is the least constant in* (1).

### Proof

From (25) we have that $\Vert uf\Vert _{q}\leq\lambda A_{p,q} \Vert \rho f'\Vert _{p}$. Taking $\lambda\rightarrow1$, we get $\Vert uf\Vert _{q}\leq A_{p,q} \Vert \rho f'\Vert _{p}$, that is, inequality (1) holds with the estimate $A_{p,q}\leq C$, which, together with (20), gives $A_{p,q}=C$. □

### Theorem 3.3

*Let* $1\leq p\leq q =\infty$. *Inequality* (1) *holds if and only if* $A_{p,q}<\infty$. *Moreover*, $A_{p,q}\leq C\leq4A_{p,q}$, *where* *C* *is the least constant in* (1).

### Proof

Let $1\leq p< q =\infty$. The necessity follows from Theorem 3.1. Let us prove the sufficiency. Let $A_{p,q}<\infty$. For $0\leq f\in \overset{\circ }{\mathrm{AC}}(I)$, we have

$$\begin{aligned} \Vert uf\Vert _{q} &=\sup_{k}\Vert uf\Vert _{q,\Delta T_{k+1}}\leq \lambda^{2}\sup_{k} \lambda^{k}\Vert u\Vert _{q,\Delta T_{k+1}} \\ &\leq \lambda^{2}\sup_{k}\lambda^{k} \Vert u\Vert _{q,T_{k+1}}\leq \lambda^{2}\sup _{k,i}\lambda^{k}\Vert u\Vert _{q,(\alpha _{i}^{k},\beta_{i}^{k})} \\ &\leq \lambda^{2} A_{p,q}\sup_{k,i} \bigl( \lambda^{pk}\bigl\Vert \rho^{-1}\bigr\Vert _{p',(c_{i}^{k},\alpha _{i}^{k})}^{-p}+\lambda^{pk} \bigl\Vert \rho^{-1}\bigr\Vert _{p',(\beta_{i}^{k},d_{i}^{k})}^{-p} \bigr)^{\frac{1}{p}} \\ &\leq \frac{\lambda^{2}}{\lambda-1} A_{p,q}\sup_{k} \bigl\Vert \rho f'\bigr\Vert _{p,\Delta T_{k}}\leq \frac{\lambda^{2}}{\lambda-1} A_{p,q}\bigl\Vert \rho f'\bigr\Vert _{p}, \end{aligned}$$

that is,

26 $$ \Vert uf\Vert _{q}\leq \inf_{\lambda>1} \frac{\lambda^{2}}{\lambda-1} A_{p,q}\bigl\Vert \rho f'\bigr\Vert _{p}=4A_{p,q}\bigl\Vert \rho f'\bigr\Vert _{p}, $$

which, as before, means that

$$C \leq4A_{p,q}. $$

Let $p=q=\infty$.

Sufficiency. From (22) we have

$$\begin{aligned}& \lambda^{k}\bigl\Vert \rho^{-1}\bigr\Vert _{1,(c_{i}^{k},\alpha _{i}^{k})}^{-1}\leq\frac{1}{\lambda-1}\bigl\Vert \rho f'\bigr\Vert _{p,(c_{i}^{k},\alpha_{i}^{k})}, \\& \lambda^{k}\bigl\Vert \rho^{-1}\bigr\Vert _{1,(\beta _{i}^{k},d_{i}^{k})}^{-1}\leq\frac{1}{\lambda-1}\bigl\Vert \rho f'\bigr\Vert _{p,(\beta_{i}^{k},d_{i}^{k})}. \end{aligned}$$

Using these relations instead of (23) and (24), we have

$$\begin{aligned} \Vert uf\Vert _{q}&\leq \lambda^{2} \sup _{k,i}\lambda^{k}\Vert u\Vert _{q,(\alpha _{i}^{k},\beta_{i}^{k})} \leq \lambda^{2} A_{p,q}\sup_{k,i} \bigl( \lambda^{k}\bigl\Vert \rho^{-1}\bigr\Vert _{1,(c_{i}^{k},\alpha _{i}^{k})}^{-1}+\lambda^{k} \bigl\Vert \rho^{-1}\bigr\Vert _{1,(\beta_{i}^{k},d_{i}^{k})}^{-1} \bigr) \\ &\leq \frac{\lambda^{2}}{\lambda-1} A_{p,q}\sup_{k} \bigl\Vert \rho f'\bigr\Vert _{p,\Delta T_{k}}\leq \frac{\lambda^{2}}{\lambda-1} A_{p,q}\bigl\Vert \rho f'\bigr\Vert _{p}. \end{aligned}$$

This gives that

$$\Vert uf\Vert _{q}\leq4A_{p,q}\bigl\Vert \rho f'\bigr\Vert _{p} $$

and

$$C\leq4A_{p,q}. $$

Necessity. Substituting the function $f_{c,h}$ into (1), we have

$$\Vert u\Vert _{q,(c-h,c+h)}\leq C \bigl(\bigl\Vert \rho ^{-1} \bigr\Vert _{1,(\alpha, c-h)}^{-1}+ \bigl\Vert \rho^{-1} \bigr\Vert _{1,(c+h,\beta)}^{-1} \bigr), $$

which means that

$$A_{p,q}\leq C. $$

Therefore,

$$A_{p,q}\leq C\leq4A_{p,q}. $$

The proof of Theorem 3.3 is complete. □

### Remark 3.3

The obtained results can be compared with the results of Theorem 8.2 of [4], where it is proved that, for $1\leq p\leq q\leq\infty$, the validity of (1) is equivalent to the condition

$$B_{p,q}=\sup_{(c,d)\subset I} \bigl[\Vert u\Vert _{q,(c,d)}, \min\bigl\{ \bigl\Vert \rho^{-1}\bigr\Vert _{p',(d,b)}, \bigl\Vert \rho^{-1}\bigr\Vert _{p',(a,c)}\bigr\} \bigr]< \infty. $$

Moreover, for the least constant *C* in (1), we have the estimates

27 $$ 2^{-\frac{1}{p}} B\leq C\leq\inf_{1< \lambda< 2}B, \quad 1\leq q< \infty, $$

and

28 $$ 2^{-\frac{1}{p}} B\leq C\leq4B, \quad q=\infty. $$

It is easy to see that $A_{p,q}< B_{p,q}$. Moreover, the estimates for the least constant *C* in (1) obtained in Theorems 3.1 and 3.3 are obviously better than in (27) and (28), respectively.

## Compactness

Let $I=(-\infty,+\infty)$ and $M=\{uf:f\in \overset{\circ }{\mathrm{AC}}(I),{∥\rho f^{\prime }∥}_{p}\leq 1\}$.

Let

$$\begin{aligned}& A^{+}_{p,q}(z)=\sup_{h>0}\frac{ (\int_{z}^{z+2h}u^{q}(t)\,dt )^{\frac{1}{q}}}{ (\Vert \rho^{-1}\Vert ^{-p}_{p',(-\infty,z)} +\Vert \rho^{-1}\Vert ^{-p}_{p',(z+2h,\infty)} )^{\frac{1}{p}}}, \\& A^{-}_{p,q}(z)=\sup_{h>0}\frac{ (\int_{z-2h}^{z}u^{q}(t)\,dt )^{\frac{1}{q}}}{ (\Vert \rho^{-1}\Vert ^{-p}_{p',(-\infty,z-2h)} +\Vert \rho^{-1}\Vert ^{-p}_{p',(z,\infty)} )^{\frac{1}{p}}}. \end{aligned}$$

### Theorem 4.1

*Let* $1\leq p\leq q<\infty$. *The set* *M* *is relatively compact in* $L_{q}(I)$ *if and only if* $A_{p,q}<\infty$ *and*

29 $$ \lim_{\vert x\vert \rightarrow \infty}A_{p,q}(x)=0. $$

### Proof

Necessity. Let *M* be relatively compact in $L_{q}(I)$. Then by Theorem 3.1 we have that $A_{p,q}<\infty$. Let $f_{c,h,\alpha ,\beta}\equiv f_{c,h}$ be the function introduced in the necessary part of Theorem 3.1.

We assume that

$$\begin{aligned}& f^{+}_{z,h,\alpha,\beta}\equiv f_{z+h,h,\alpha,\beta}, \quad\quad f^{-}_{z,h,\alpha ,\beta}\equiv f_{z-h,h,\alpha,\beta}, \\& g^{+}_{z,h,\alpha,\beta}(t)=f^{+}_{z,h,\alpha,\beta}(t) \bigl(\bigl\Vert \rho^{-1}\bigr\Vert _{p',(\alpha,z)}^{-p}+ \bigl\Vert \rho^{-1}\bigr\Vert _{p',(z+2h,\beta)}^{-p} \bigr)^{-\frac{1}{p}}, \\& g^{-}_{z,h,\alpha,\beta}(t)=f^{-}_{z,h,\alpha,\beta}(t) \bigl(\bigl\Vert \rho^{-1}\bigr\Vert _{p',(\alpha,z-2h)}^{-p}+ \bigl\Vert \rho^{-1}\bigr\Vert _{p',(z,\beta)}^{-p} \bigr)^{-\frac{1}{p}}. \end{aligned}$$

Since $g_{z,h,\alpha ,\beta }^{\pm }\in \overset{\circ }{\mathrm{AC}}(I)$ and $\Vert \rho(g^{\pm}_{z,h,\alpha,\beta})'\Vert _{p}\leq1$, we have $g^{\pm}_{z,h,\alpha,\beta}\in M$, and by the Frechet-Kolmogorov theorem [6], p.10 we get

$$\begin{aligned} 0&=\lim_{N\rightarrow\infty}\sup_{f\in M} \biggl( \int_{\vert t\vert >N}\vert uf\vert ^{q}\,dt \biggr)^{\frac{1}{q}}\geq \lim_{N\rightarrow\infty}\sup_{z,h,\alpha,\beta} \biggl( \int _{t>N}\bigl\vert ug^{+}_{z,h,\alpha,\beta}\bigr\vert ^{q}\,dt \biggr)^{\frac{1}{q}} \\ & \geq \lim_{N\rightarrow\infty}\sup_{z>N}\sup _{h>0} \frac{ (\int_{z}^{z+2h}u^{q}(t)\,dt )^{\frac{1}{q}}}{ (\Vert \rho^{-1}\Vert _{p',(-\infty,z)}^{-p}+ \Vert \rho^{-1}\Vert _{p',(z+2h,\infty)}^{-p} )^{\frac {1}{p}}}=\lim_{z\rightarrow\infty}\sup A^{+}_{p,q}(z). \end{aligned}$$

Therefore,

30 $$ \lim_{z\rightarrow\infty}A^{+}_{p,q}(z)=0. $$

Similarly, working with the function $g^{-}_{z,h,\alpha,\beta}$, we get $\lim_{z\rightarrow-\infty}A^{-}_{p,q}(z)=0$, which, together with (30), gives (29).

Sufficiency. Let $A_{p,q}<\infty$ and (29) hold. Then, on the basis of Theorem 3.1, the set *M* is bounded in $L_{q}(I)$. Therefore, by the Frechet-Kolmogorov theorem it suffices to show that

31 $$ \lim_{N\rightarrow\infty}\sup_{f\in M} \biggl( \int_{\vert t\vert >N}\vert uf\vert ^{q}\, dt \biggr)^{\frac{1}{q}}=0. $$

Let

$$\widehat{u}(t)=\left \{ \textstyle\begin{array}{l@{\quad}l} u(t), & t< N, \\ 0, & t\geq N, \end{array}\displaystyle \right . \quad \mbox{and}\quad \widetilde{u}(t)=\left \{ \textstyle\begin{array}{l@{\quad}l} 0, & t\leq N, \\ u(t), & t>N. \end{array}\displaystyle \right . $$

Hence, by Theorem 3.1 we have

$$\begin{aligned}& \biggl( \int_{-\infty}^{\infty} \vert \widehat{u}f\vert ^{q}\, dt \biggr)^{\frac{1}{q}}\leq f(\lambda_{1})\sup _{x< N}A_{p,q}(x) \quad \mbox{for } f\in M, \\& \biggl( \int_{-\infty}^{\infty} \vert \widetilde{u}f\vert ^{q}\, dt \biggr)^{\frac{1}{q}}\leq f(\lambda_{1})\sup _{x>N}A_{p,q}(x)\quad \mbox{for } f\in M. \end{aligned}$$

Then

$$\sup_{f\in M} \biggl( \int_{\vert t\vert >N}\vert uf\vert ^{q}\,dt \biggr)^{\frac{1}{q}}\leq f(\lambda_{1})\sup_{\vert x\vert >N}A_{p,q}(x). $$

This, together with (29), gives (31). The proof of Theorem 4.1 is complete. □
